# Computational modeling reveals biological mechanisms underlying the whisker flick EEG

**DOI:** 10.1016/j.isci.2025.113793

**Published:** 2025-10-17

**Authors:** Joseph Tharayil, James B. Isbister, Esra Neufeld, Michael Reimann

**Affiliations:** 1 Blue Brain Project, École Polytechnique Fédérale de Lausanne (EPFL) Campus Biotech, 1202 Geneva, Switzerland; 2 IT’IS Foundation, 8004 Zurich, Switzerland

**Keywords:** systems neuroscience, sensory neuroscience, signal processing, modeling signal processing system, computer simulation

## Abstract

Whisker flick stimulation is a commonly used protocol to investigate somatosensory processing in rodents. Neural activity evoked by whisker flicks produces a characteristic electroencephalography (EEG) waveform known as a somatosensory evoked potential. In this paper, we use computational modeling to make predictions about the neural populations that contribute to this signal, either through their own membrane currents or the membrane currents they elicit in downstream populations. While the model cannot fully explain the mechanisms of somatosensory evoked potential (SEP) generation, we predict that the initial positive deflection of the EEG waveform is driven largely by direct thalamic inputs to layer 2/3 and layer 5 pyramidal cells, while the negative deflection is driven by a more complex mix of sources, including thalamic and recurrent cortical connectivity. Small changes to the local connectivity of the circuit can have an important impact on the recorded EEG, without substantially affecting firing rates, suggesting that EEG may be useful in constraining *in silico* neural models.

## Highlights

• The BBP’s somatosensory cortex model replicates key features of *in vivo* whisker flick EEG
• We predict neural populations’ contributions to different components of the EEG
• Changes to circuit connectivity have large impacts on EEG without changing firing rates

## Graphical abstract

**Figure:**
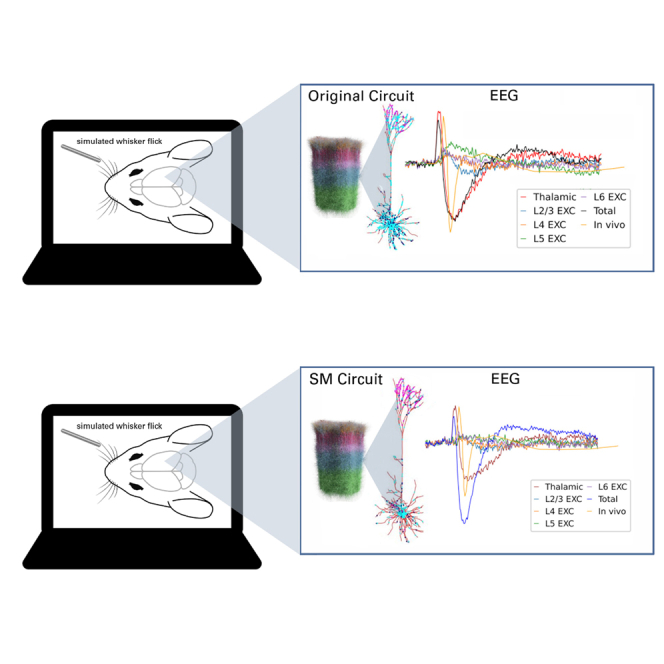

## Introduction

The somatosensory evoked potential (SEP) is the electroencephalography (EEG) signal evoked by somatosensory stimuli, recorded at the skull. SEPs are commonly studied in the context of the whisker flick paradigm in rodents for the study of sensory processing, integration, and plasticity.^1^ Understanding the whisker flick SEP can provide insight into the biophysical basis of somatosensory processing and the related EEG. Following a whisker flick, the triggered SEP has a stereotypical waveform with stimulus intensity-dependent amplitude, consisting of a positive deflection (P1) with a width of around 5 ms, immediately followed by a negative deflection (N1) of the same width.^2^ It is believed^2^ that the rise of the P1 component is driven by the activation of thalamocortical synapses, but the cause of its decay is not fully understood. In contrast to the view that P1 is fully thalamocortically driven, the P1 component does not manifest in the EEG signal until around postnatal day 13 (P13), suggesting that the signal arises due to recurrent activity in supragranular layers that appears with maturation of local connectivity^3^ and cannot be explained solely by thalamic input. However, the local connectivity patterns that permit the emergence of the P1 component are not known.

Experimental studies^2^ have shown that blocking inhibitory synaptic activity alters the shape of the late phase of the N1 component but does not affect the P1 component. However, the degree to which this effect can be attributed directly to decreased inhibitory post-synaptic currents in pyramidal neurons, as opposed to broader changes in circuit-level activity, is unclear. Modulation of the GABA_A_ receptor has been shown to cause changes to the voltage-sensitive dye signal that depend at least in part on changes in disynaptic inhibition.^4^ Computational modeling at the circuit level may be very useful in understanding the impact of inhibition on the N1 component of the SEP.

**Figure 1:**
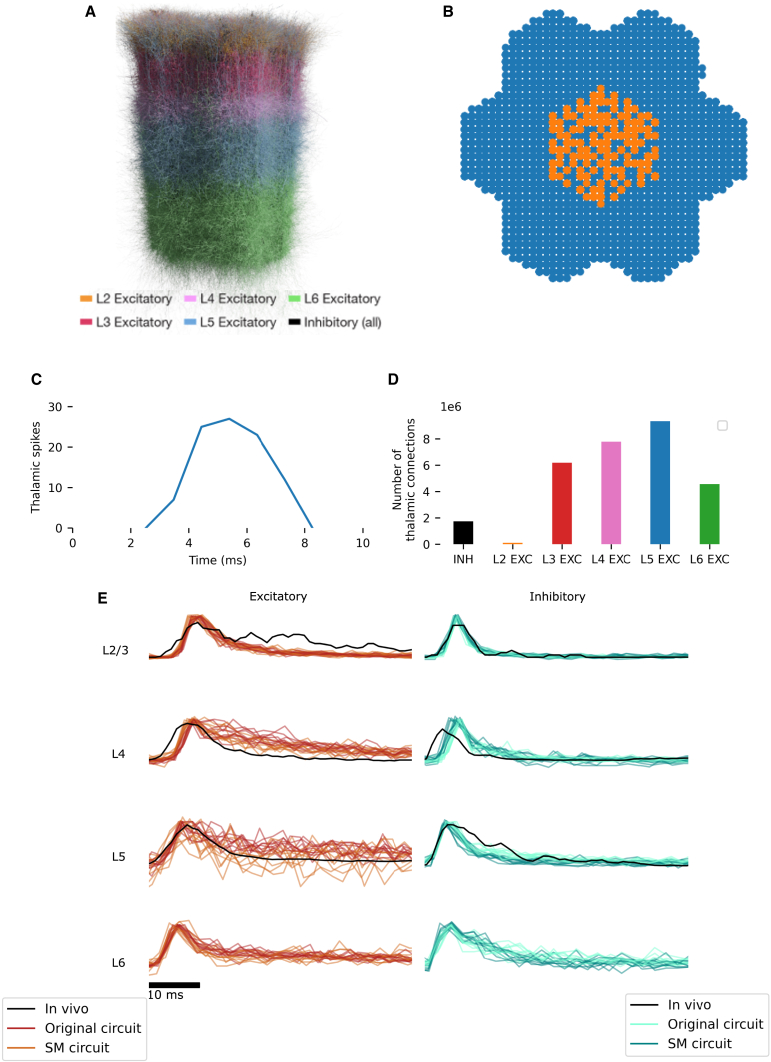
Overview of *in silico* circuit (A) Seven column subvolume of nbS1 used in this study. (B) Whisker flick is simulated by activating a subset of thalamic fibers innervating the cortex. Activated fibers are marked in orange. Fibers not activated are marked in blue. (C and D) Thalamic spike times are drawn from an *in vivo* PSTH recorded in response to whisker flick.^7^ (D) Thalamic innervation of nbS1 primarily targets excitatory populations. (E) PSTHs from both the original and the SM circuits in response to simulated whisker flick closely match *in vivo* PSTHs. All PSTHs are baseline- and max-normalized, and *in vivo* PSTHs are smoothed. All PSTHs are zero-indexed to the onset of spiking activity (defined as overall firing rate exceeding the baseline by 3 SDs) in the respective condition (i.e., calculated separately for the *in vivo* experiment and the original and SM circuits).

**Figure 2:**
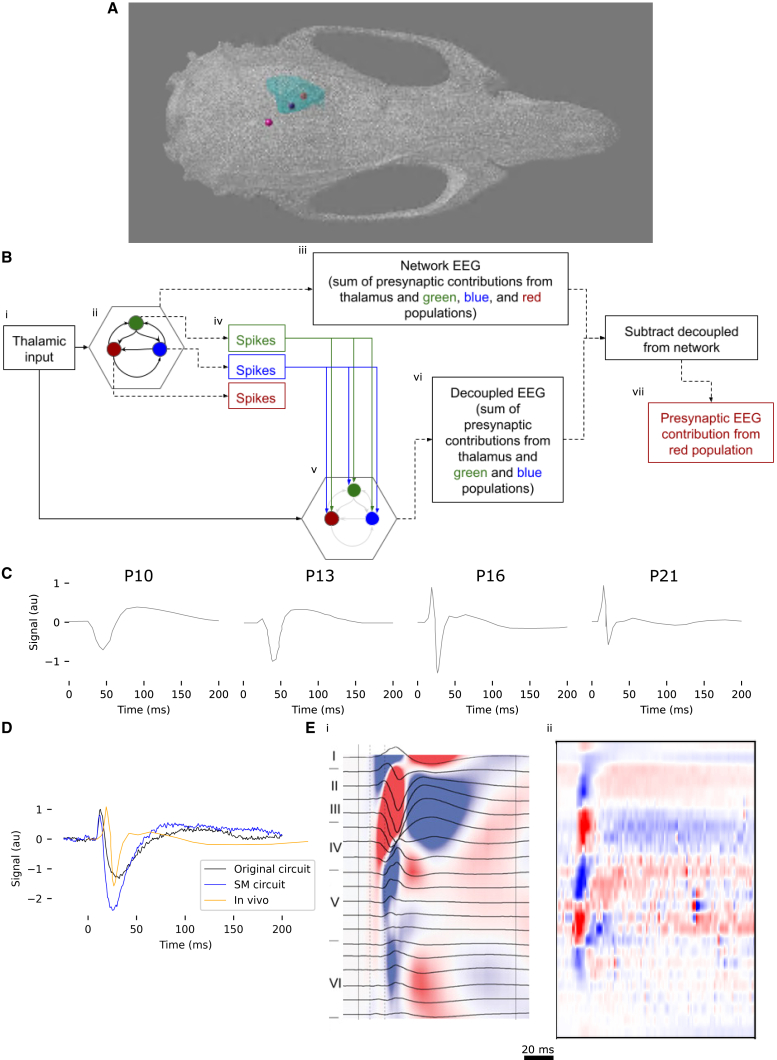
Experimental procedure and overview of results (A) Partial visualization of the finite element model used in this study. Skull (white) and somatosensory cortex (light blue) are shown. EEG electrodes are modeled as small spheres implanted in the skull over the forelimb region of the somatosensory cortex (red), the hindlimb region of the somatosensory cortex (dark blue), and the lambda point of the skull (pink). (B) Cartoon of the workflow used to isolate the contribution of a specified presynaptic population to the SEP. (i and ii) Original simulation. Thalamic spikes (i) are played into a circuit (ii, hexagon) with several interacting populations (for clarity, only three are shown). (iii) The EEG calculated from the circuit is referred to as the “network EEG.” (iv and v) To calculate the contribution to the EEG from the population represented by the red circle, the spikes from the other two populations are recorded in the original simulation (iv), and these populations are artificially made to fire. The efferent synapses of these populations are artificially activated at the same times as when they fire in the original simulation (iv and v, blue and green arrows), in a circuit (v) in which additional spikes generated by activity in the new simulation are not transmitted by efferent synapses (grayed-out arrows represent deactivated synaptic connections). (vi) The EEG recorded from this simulation is referred to as the “decoupled EEG.” (vii) The decoupled EEG is subtracted from the network EEG to obtain the contribution of the population represented by the red circle. (C) *In vivo* SEPs recorded at various ages from P10 to P21, digitized from Quairiaux et al.^3^ and normalized to the peak P1 amplitude on P16. (D) Comparison of SEP signals from the original and SM circuits with *in vivo* data obtained on P16.^3^ The *in vivo* signal and the *in silico* signal from the original circuit are normalized to their respective P1 peaks; the signal from the SM circuit is normalized to the P1 peak from the original circuit. (E) CSD recorded (i) *in vivo* on P21 (Reproduced with permission from Quairiaux et al.^3^) and (ii) in the original circuit model. Horizontal bars to the left of the traces indicate boundaries between different cortical layers. Note that *in vivo* and *in silico* traces do not necessarily align due to differences in electrode spacing and layer boundary estimation.

The Blue Brain Project (BBP) model of the rat non-barrel primary somatosensory cortex (nbS1) consists of ∼4.2 million reconstructed neurons with accurate morphologies, optimized physiological properties, and algorithmically generated connectivity.^5^^,^^6^ In this study, we simulate a subvolume of the BBP nbS1 model with ∼210,000 neurons (Figure 1A), which has been shown to produce realistic firing activities in response to a simulated whisker flick stimulus modeled by activating virtual thalamic fibers that innervate the cortical model (Figures 1B–1D).^6^ The peri-stimulus-time histograms (PSTHs) of firing in layer-wise excitatory/inhibitory populations closely match *in vivo* data (Figure 1E),^6^ thus implying a high-level of precision in the population responses studied here. In this paper, we study two different versions of this model, which have somewhat different cortico-cortical connectivity. We refer to the first version of the model reported in Isbister et al.^6^ as the “original” circuit and the second version, with rewired connectivity (see below), as the “Schneider-Mizell” or SM circuit (Figures 1E and 2D). We refer to both variants of the neural circuit model, collectively, as nbS1.

To simulate the EEG signals associated with our whisker flick stimulus simulation, we use a detailed finite element electromagnetic model of the rat head in conjunction with BlueRecording,^8^ a set of software tools which enables the simulation of extracellular recordings from neural circuit models by calculating a “weights file” that specifies the contribution weights of transmembrane currents from each compartment in the model to the signal.

In particular, we aim to identify the contributions of different neural populations (separated into 6 cytological layers, denoted L1 to L6, and excitatory and inhibitory (EXC and INH) cell types) to the SEP in our model, both through their own transmembrane currents (i.e., a population’s “postsynaptic contribution”) and the transmembrane currents they elicit in downstream populations through efferent synaptic activation (i.e., a population’s “presynaptic contribution”). The latter does not include multi-synaptic effects. We refer to the contribution of a presynaptic-postsynaptic pathway (i.e., the contribution of L5INH-L4) to denote the contribution to the SEP of the currents in the postsynaptic population elicited by the synaptic activation of the presynaptic population.

While disambiguating the contributions of specific postsynaptic populations would be effectively impossible *in vivo*, doing so is trivial *in silico*. BlueRecording reports the contribution of each neuron in the model to the SEP; these neuronal contributions are summed in postprocessing to generate population contributions.^8^ In this paper, we report the postsynaptic contributions of layers 2/3, 4, 5, and 6; this is effectively equivalent to the contribution of excitatory postsynaptic cells in the corresponding layers, as the contributions of inhibitory cells are negligible (Figure S1).

To isolate the presynaptic contribution of a specified population (Figure 2B, red circles) to the EEG, we want to remove the transmembrane currents elicited by the population of interest in downstream populations through efferent synaptic activation. To approximate this, we first simulate the circuit as usual (Figure 2Bi and Bii) and calculate the resulting EEG, which we refer to as the “network EEG” (Figure 2Biii). We then run a new simulation (Figure 2Bv) in which (1) the efferent synapses of neurons (Figure 2, blue and green circles) outside of the population of interest are artificially activated (Figures 2Biv and Bv, blue and green arrows) at the times they spiked in the original simulation (Figure 2Biv), (2) additional spikes generated by activity in the new simulation are not transmitted by efferent synapses (Figure 2Bv, gray arrows), and (3) spikes from the population of interest are not transmitted by efferent synapses. The “decoupled EEG” (Figure 2Bvi) calculated from this simulation is thus driven by the same currents as the network EEG, excluding the transmembrane currents elicited by the population of interest in downstream populations through efferent synaptic activation. The presynaptic contribution of the population of interest is, therefore, given by the difference between the network EEG calculated from the original simulation and the decoupled EEG.

One cannot isolate the impacts of particular cortico-cortical pathways by simply setting connection weights between the populations of interest to zero and subtracting the resulting SEP from the original signal, as this would have unpredictable effects on the firing rates of the postsynaptic population, and consequently on downstream populations. It would, therefore, be impossible to disambiguate the effects of the removed synaptic inputs from the broader changes in circuit activity, which may well deviate unrealistically from *in vivo* conditions. The problem has been previously approached with a similar solution to ours, namely, replaying only the spikes from the presynaptic population of interest,^9^ but we believe that our approach is more informative because the approach in Rimehaug et al.^9^ removes the majority of spikes, which could potentially have significant effect on the membrane dynamics of the postsynaptic neurons (which in normal conditions are highly leaky), thus affecting the SEP.

**Figure 3:**
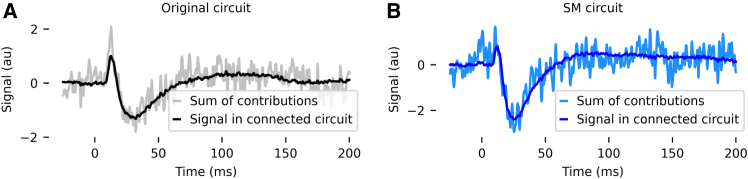
Validation of calculation of population contributions **(**A) SEP from the original circuit, compared to the sum of the calculated contributions from each cortical population and the thalamic input. (B) SEP from the SM circuit, compared to the sum of the calculated contributions from each cortical population and the thalamic input.

In principle, it is possible that even our procedure of disconnecting the circuit and playing in the majority of the spikes from the connected simulation could lead to an entirely different dynamical regime. If this were the case, the procedure would not produce accurate estimates of the contributions of the presynaptic populations. However, as the sum of the contributions of all presynaptic populations approximates the original signal (Figure 3), it is unlikely that the procedure places the network in a very different dynamical regime.

The approach we implement in this paper allows us to predict the presynaptic contributions of various populations in the circuit. However, that contribution can vary depending on the locations of synapses with respect to postsynaptic morphologies. For example, Teleńczuk et al.^10^ predict strong unitary local field potentials of inhibitory spikes in the hippocampus due to their perisomatic locations. To explore the impact of synapse location on the EEG signals produced in our simulations, we calculate the SEP from a rewired version of the somatosensory cortex model,^11^ referred to as the SM circuit, which incorporates more accurate local connectivity obtained from the MiCrONS electron microscopy dataset.^12^ In particular, synapses from layer 5 large basket cells and layer 5 nest basket cells on to layer 5 pyramidal cells (PCs) are moved closer to the soma (Figure 6F). We have previously shown^6^ that the SM connectome produces substantially identical firing rates compared to the original model, after recalibration of the input noise (cf. Figure 1E).

We compare the SEPs generated *in silico* to *in vivo* results from Quairiaux et al.^3^ (Figure 2D). In particular, we are interested in how our model compares to *in vivo* data recorded on P16, as our model is based largely on data obtained from an animal on P14, with additional data from older animals. Because our model encompasses only a small subset of the somatosensory cortex, we cannot expect the amplitude of the *in silico* SEP to match that of the *in vivo* signal (cf. section model EEG and LFP amplitudes are self-consistent). We, therefore, normalize both the *in vivo* signal and the *in silico* signals from the original circuit to their respective P1 peaks, comparing only the shape of the signals. The *in silico* signal from the SM circuit is normalized to the P1 peak of the original circuit.

## Results

### Sum of estimated presynaptic contributions approximates total signal

To validate the approach of using spike replay to isolate the contributions of individual cortical populations to the SEP, we compare the SEP generated by the fully connected circuit to the sum of the presynaptic contributions of each neural population (including the cortical populations and the contribution of the thalamic input). For both the original and the SM circuits, the sum of the population contributions does approximate the original signal (Figure 3). However, much more high-frequency noise is present in the reconstructed signal than in the original signal. This can be explained by differences in spike timing between the network (Figure 2Bii) and the decoupled (Figure 2Bv) simulations. This difference in spike timing occurs because synaptic currents from the population of interest (the population whose presynaptic contribution we wish to calculate, i.e., the red population in Figure 2B) are not present in the decoupled network, which naturally affects the firing of the postsynaptic populations. This difference in firing means that the decoupled EEG will differ from the network EEG not only because of the loss of the contribution of these synaptic currents and the associated return currents but also due to differences in the contributions of active currents. When the decoupled EEG is subtracted from network EEG to calculate the contribution of the presynaptic population, high-frequency noise results from the differences in the timing of the action potentials’ contribution to the signal; this noise is amplified as the contributions of the presynaptic populations are summed.

### SEPs modeled *in silico* approximate *in vivo* recordings

We compare the SEPs produced by our circuit models to *in vivo* SEPs from Quairiaux et al.^3^ (cf. Figure 2). The recording electrode is implanted in the skull above the somatosensory cortex. We note that the *in vivo* recordings were conducted with a reference electrode over the lambda point (the point along the midline of the skull where the occipital bone meets the two parietal bones), while *in silico* recordings were conducted with a reference over the hindlimb region (Figure 2A). However, we show that changing the reference location only affects the SEP by a linear scaling factor (Figure S2).

As our model is based primarily on data from P14, with some data obtained from older animals, we expect the associated *in silico* SEPs to most closely resemble *in vivo* SEPs from P16. This is indeed the case; like the *in vivo* SEP on P16, but unlike those from other developmental time points, our *in silico* SEP has a prominent P1 peak, followed by a somewhat larger N1 peak (Figures 2C and 2D). Hence, in the remainder of this paper, we exclusively compare our *in silico* results to *in vivo* results from P16.

We then find that the relative N1 amplitude of the *in vivo* SEP also matches that of the original *in silico* SEP (Figure 2D). However, the P1 component *in vivo* occurs approximately 5 ms later than *in silico*, and the relative amplitude of the N1 component of the SEP from the SM circuit is substantially greater than *in vivo* (Figure 2D). The width of the N1 component *in silico* is roughly three times that of the *in vivo* SEP, although the SM circuit produces an N1 component 25% narrower than the original.

Our results indicate that the model successfully replicates the key features of the SEP. However, the duration of the simulated P1 component is a better match to the *in vivo* data than that of the N1 component. The observation that the width of the N1 is narrower in the SM circuit than in the original, while the amplitude is larger in the SM circuit, suggests that this rewiring has mixed effects on the realism of our model (cf. sections perisomatic targeting explains the difference between the original and the SM circuit and specificity of layer 5 inhibition drives differences in width of the N1 component in silico; Figures 6 and 7).

In addition to the SEP, we compare the current source density (CSD) recorded *in vivo* on P21 to the CSD recorded in our original circuit model (due to limitations on computational resources, we do not calculate CSD from the SM circuit; cf. sec. method details). As in the original data, our model produces a large current sink extending from L4 to L3, flanked by two sources, one extending from L3 to L1 and the other from L4 to L5 (Figure 2E). However, the current sink in our model does not extend as far upward as that recorded *in vivo*. Both in our model and *in vivo*, the L3/4 sink is followed by a current source approximately 20 ms after the stimulus; however, the current source *in vivo* is larger in amplitude and more temporally precise than in our model. In our model, we observe a second large current sink at the bottom of L5 (Figure 2Eii), flanked by the aforementioned L4/5 source and a source in L6. In the *in vivo* data, the L5 current sink does not appear; rather, the sources in L4/5 and in L6 are separated by a region of low current density (Figure 2Ei). Our model thus broadly replicates the *in vivo* CSD, albeit with the addition of a large early current sink in L5.

### Model EEG and LFP amplitudes are self-consistent

**Figure 4:**
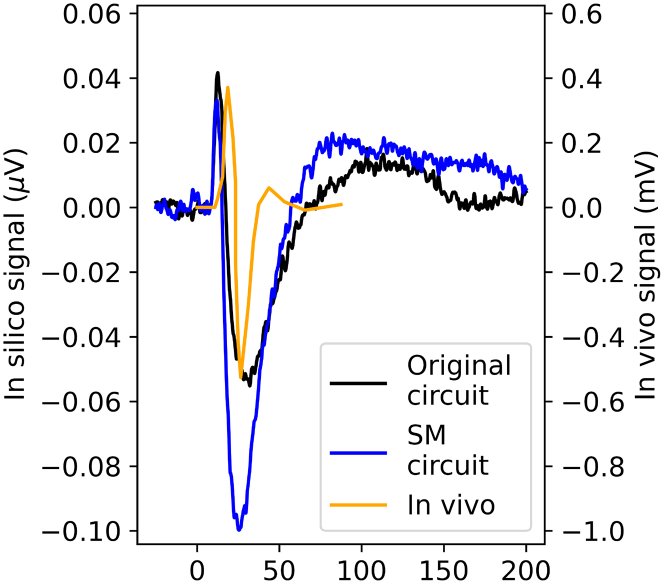
Comparison of raw EEG amplitudes *In vivo* EEG amplitudes are 10^4^× greater than *in silico*.

While our model successfully replicates the shape of the *in vivo* SEP, it does not capture its amplitude: While reported *in vivo* P1 component amplitudes range from 40 *μm*V^13^^,^^14^^,^^15^ to 750 *μm*V,^3^ the *in silico* SEP of the original circuit only reaches 0.1 *μm*V (Figure 4). While the EEG amplitudes produced by our model are thus a factor 10^4^ smaller than the reference *in vivo* data from Quairiaux et al.,^3^ there is more than an order of magnitude variation in reported *in vivo* whisker flick EEG amplitudes; the present model only captures a subregion of the somatosensory cortex, and as discussed in the section "differences between in silico and in vivo signals," there is uncertainty about the correct extent of whisker flick input to be applied to the model.

Hence, we focus on ensuring that our model is internally consistent in terms of the local field potentials (LFPs), CSD distribution, and EEG signal, rather than a quantitative comparison with literature data. Our model produces LFPs with peak amplitude of 0.18 mV. This is approximately a factor 5–7 lower than typical whisker flick LFP amplitudes reported in the literature,^3^^,^^16^^,^^17^ although Riera et al.^13^ report LFP magnitudes more comparable to ours. Based on the CSD (Figure 2Eii) derived from our *in silico* LFP recordings, we estimate a dipole moment density (with respect to cross-sectional area) of 0.023 *nAm*/*mm*^2^, by integrating the CSD over the depth of the column at the time of the peak current density (see method details). For this, we assume that the LFP is produced by a cortical region with a diameter of 1 mm (i.e., one column and half of each of the neighboring columns). Previous estimates of dipole density assumed that the LFP signal is produced by a single column^18^; however, experiments have shown that neighboring columns produce substantial current densities.^16^

With an average dipole-aligned lead field of ∼4 *V* /*mm*/*A* (cf. Figure 6E) in the central column, we estimate a contribution of 1.85 × 10^−8^*V* to the EEG from the central column, by scaling the dipole density by the surface area of the column and multiplying with the lead field. This results in an estimated peak circuit EEG amplitude of 1.3 × 10^−7^*V*, which is comparable to the EEG of 0.1 *μm*V obtained using BlueRecording.

### The *in silico* P1 component is mostly driven by thalamic input

**Figure 5:**
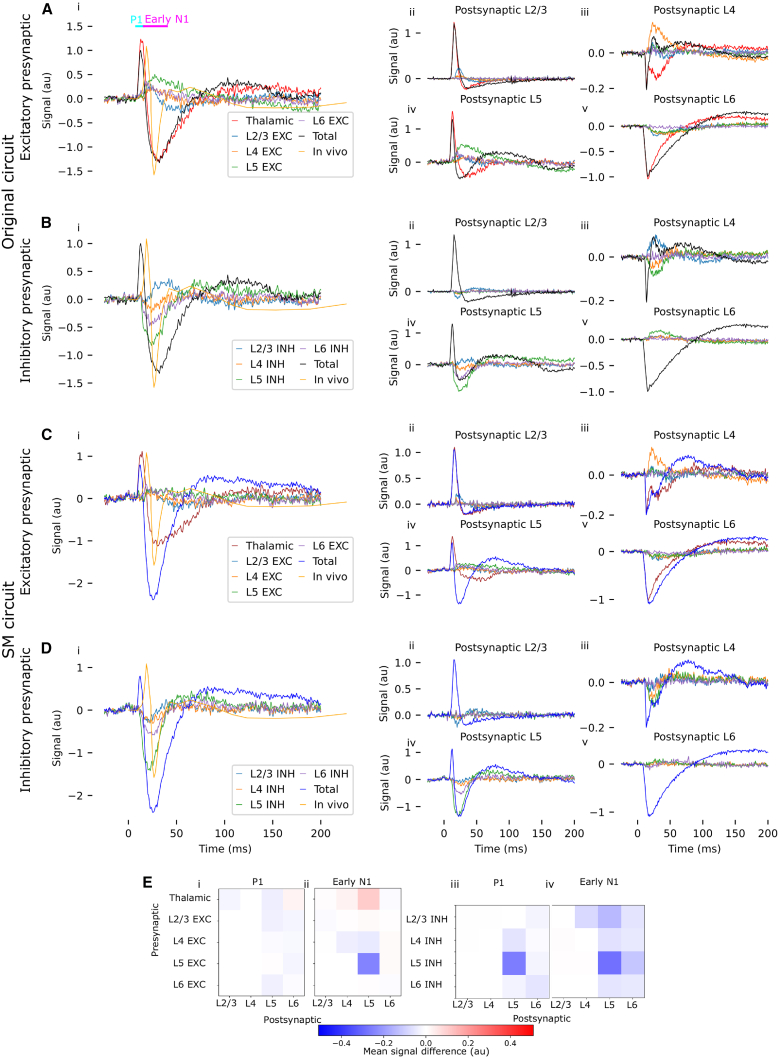
Contributions of neural populations to the EEG (Ai) SEP from the original circuit, and the presynaptic contributions to the SEP of the different excitatory populations. Timings of the P1 component (defined as the period from 8 to 16 ms post-stimulus) and the early N1 component (defined as the period from 16 to 40 ms post-stimulus) are marked with blue and pink bars, respectively. (Aii–v) Contributions of the different presynaptic-postsynaptic pathways to the SEP, for excitatory presynaptic populations (traces) and all postsynaptic layers (panels). (B) Same as (A), but for inhibitory presynaptic populations. (C) Same as (A), but for the SM model. (D) Same as (B), but for the SM model. (E) Differences between the SM and the original circuit in the mean postsynaptic contribution from the different layers, attributable to presynaptic input from corresponding populations.

In our original circuit model, the isolated presynaptic thalamic contribution to the SEP (Figure 5Ai, red trace) is quite similar to the total SEP obtained from the original circuit (Figure 5Ai, black trace). (We assume that the thalamic fibers themselves have no postsynaptic contribution to the EEG, as only their axon terminals are located in the cortex, but cf. section limitations of the study). However, the presynaptic thalamic contribution produces a slightly larger P1 component than the total SEP. The difference in the P1 component can be attributed to the smaller postsynaptic contribution from L2/3 (Figure 5Aii, black trace) and, particularly, L5 (Figure 5Aiv, black trace) in the circuit, compared to the postsynaptic contribution from these layers evoked by the presynaptic thalamic contribution alone (red traces in Figure 5Aii and Aiv). We also note that the postsynaptic contribution from layer 4 in the connected circuit has a sharp negative peak during the P1 component (Figure 5Aiii, black trace); however, the impact thereof on the overall signal is limited, as the postsynaptic layer 4 contribution is an order of magnitude smaller than that of other layers (see section postsynaptic contributions from L4 are small compared to other layers).

**Figure 6:**
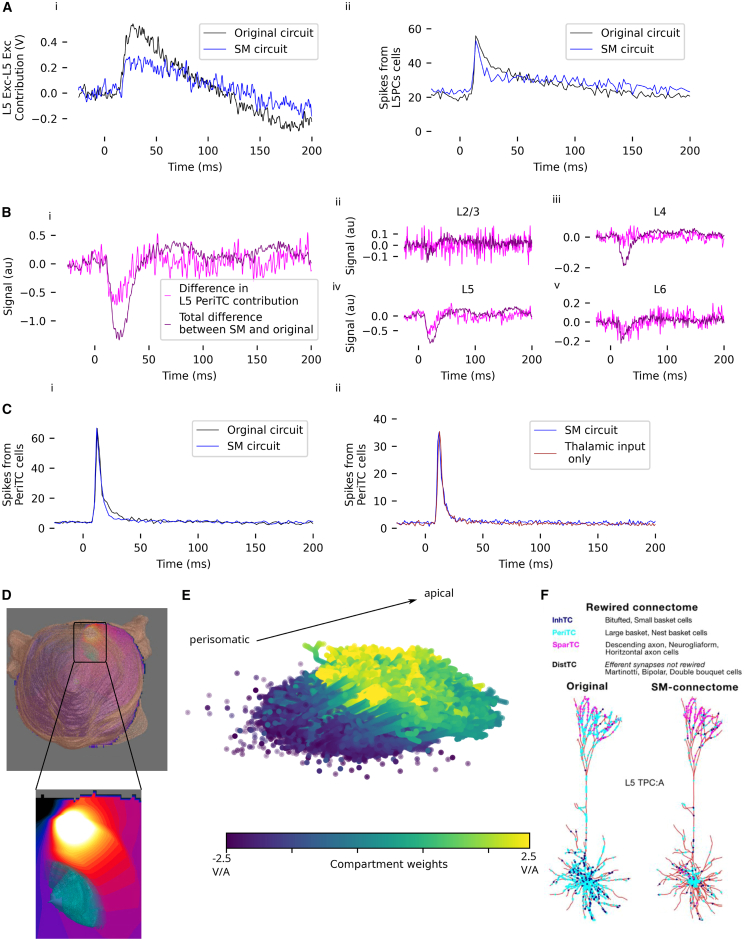
Explanation of differences between EEG from original and SM circuits (A) (i) Contribution of L5-L5 excitation to the SEP in the original and SM circuits. (ii) Firing rates of layer 5 pyramidal cells in the original and SM circuits. (B) (i) The difference in contribution to the SEP due to presynaptic excitatory input from L5 perisomatic targeting cells accounts for the difference between the SM and the original circuit. (ii–v) Differences in the postsynaptic contribution from each layer, attributable to L5 PeriTC presynaptic input. Note the difference in *y* axis scales between panels—the *y* axis range for subpanel (iv) is ∼5 times greater than that for the other subpanels. (C) (i) Firing rates for layer 5 PeriTC cells do not differ substantially between the original and the SM circuit. (ii) Firing rates for layer 5 PeriTC cells do not differ substantially between the SM and the disconnected circuit. (D) Potential induced by current applied between recording electrodes in a finite element model of the rat head. Skin visualized in beige. Zoom: Potential over the somatosensory cortex (visualized in light blue). (E) Sensitivity of the EEG signal to currents over the membrane of layer 5 pyramidal cell morphologies. Each electrical compartment of a neuron is indicated as a dot at its center; the sensitivity is reflected in its color. (F) Rewiring moves PeriTC synapses on pyramidal cells to perisomatic compartments (Reproduced from Isbister et al.^6^ under a CC-BY 4.0 license); “SM-connectome” indicates the SM circuit.

In addition to the original model, we also simulate the SM circuit. In the SM circuit, parvalbumin-positive interneurons target perisomatic regions with more specificity than in the original circuit (Figure 6F, see Reimann et al.^5^ for details). As in the original circuit, the total P1 component in the SM circuit (Figure 5Ci, dark blue trace) is smaller than what would be evoked by the presynaptic thalamic contribution alone (Figure 5Ci, red trace), due primarily to a smaller postsynaptic contribution from layer 5 in the total SEP (Figure 5Civ, dark blue trace) than what would be evoked by the thalamic input alone (Figure 5Civ, red trace).

The difference between the total signal and the contribution of thalamic input to the signal is evidently the contribution of recurrent connectivity within the circuit. We further split this contribution into the individual presynaptic and postsynaptic contributions from each population (see STAR Methods). Contributions from layer 1 are not shown, as they are very small (see Figure 3; section sum of estimated presynaptic contributions approximates total signal for why the sum of the presynaptic contributions does not add up perfectly to the total signal).

In the original circuit, the reduction in the amplitude of the P1 component by recurrent connectivity—i.e., comparing the SEP produced by the circuit (black trace in Figure 5Ai) to the SEP produced by presynaptic thalamic input alone (red trace in Figure 5Ai)—can be attributed to a negative contribution to the SEP from L5-L5 inhibition (green trace in Figure 5Biv). The same holds for the SM circuit (green trace in Figure 5Div). The reduction in the amplitude of P1 due to L5-L5 inhibition is stronger in the SM circuit than in the original (Presynaptic L5INH-Postsynaptic L5 in Figure 5E). However, as recurrent activity only changes the amplitude of the P1 component by ∼15%, the primary contributor to P1 is clearly the thalamic input.

### L5-L5 inhibition has a significant impact on N1 *in silico*

In the original circuit, the total N1 component (Figure 5Ai, black trace) is similar to that produced by the thalamic input alone (Figure 5Ai, red trace). Unlike for the P1 component, however, this does not result from a dominating presynaptic thalamic contribution to the N1. While presynaptic thalamic inputs to layer 6 substantially contribute to the N1 (red traces in Figure 5Av), and in particular prescribe the time course of the recovery, thalamic inputs do not dominate as they do in the case of the P1 component. We observe that during the early part of the N1 component (prior to ∼40 ms post-stimulus), presynaptic inhibitory input from L5 has a strong driving effect on the postsynaptic contribution of L5 to the N1 component (green trace in Figure 5Biv), as does, to a lesser extent, inhibition from L6 to L5 (red trace in Figure 5Biv). However, this is partially compensated for by the effect of cortico-cortical excitation, particularly L5 excitation to L5 (green trace in Figure 5Aiv), which has the opposite effect on the early N1.

Compared to the original circuit, the SM circuit has a significantly stronger N1 component (dark blue trace in Figures 5C and 5D), due to a more negative postsynaptic contribution from L5 cells (dark blue trace in Figure 5Div). This is explained by a weaker (but still positive) presynaptic contribution of L5 excitatory cells to the early N1, and by a more negative presynaptic contribution of L5 inhibitory cells (Figure 5E). As for the original circuit, the early N1 component in the SM circuit is driven primarily by L5-L5 inhibition (green trace in Figure 5Di), with a smaller role for L6-L5 inhibition (red trace in Figure 5Di).

We observe that the postsynaptic contribution of layer 4 during the early N1 component is strongly affected by recurrent connectivity, both in the original circuit (Figure 5Aiii, black trace) and in the SM circuit (Figure 5Ciii, dark blue trace). In both cases, this difference can be attributed primarily to the effect of L4-L4 excitation (orange traces in Figures 5Aiii and 5Ciii). However, this has a much smaller effect on the N1 component than recurrent connectivity in layer 5, since the magnitude of the postsynaptic layer 4 contribution to the EEG is an order of magnitude smaller than that of layer 5 (see section postsynaptic contributions from L4 are small compared to other layers).

### Postsynaptic contributions from L4 are small compared to other layers

We observed that the contribution of postsynaptic layer 4 cells to the EEG is an order of magnitude smaller than that of other layers (Figure 5). Factors influencing the magnitude of the contribution from a postsynaptic population include the number of cells in the population, the correlation in the contribution of individual cells, and the magnitude of the contribution from each cell. The magnitude of the cellular contribution is in turn influenced by the range of the compartment weights over the cell (a larger range of weights leads to a larger contribution), the alignment of synaptic and return currents with the axis over which the weights vary, and the amplitude of the currents themselves.

For a random sample of neurons in each population, we calculate the average range in compartment weights over the neuron (difference between 90th and 10th percentiles). The range of weights of layer 5 cells is, on average, more than twice that of L2/3 and L4 cells (Table 1). However, the L2/3 population is larger than L4 and L5. Were these factors to scale proportionally, we would expect L2/3 and L4 to have similar contribution magnitudes and that of L5 to be ∼2 times larger. The observed contribution from layer 4 is, therefore, smaller than expected. The whisker flick stimulus effectively synchronizes activity between neurons,^6^ which would tend to increase the amplitude of the EEG contribution. Therefore, we speculate that this difference can instead be attributed to a lower amplitude of current arriving in L4, or to these currents not aligning with the axis of the weights over L4.

**Table 1:** Factors influencing postsynaptic contributions for selected populations

Population	Cell count	Weights range (V/nA)	Peak-to-peak SEP amplitude (V)
L2/3	53,000	1.2 × 10^−9^	4.7 × 10^−8^
L4	29,000	1.5 × 10^−9^	8.6 × 10^−9^
L5	35,000	3.1 × 10^−9^	7.6 × 10^−8^

### Perisomatic targeting explains the difference between the original and the SM circuit

We found that the contribution of L5-L5 excitation to the SEP is greater in the original than in the SM circuit (Figures 5E and 6Ai). While firing rates in layer 5 pyramidal cells do have a narrower peak and longer tail in the SM circuit than the original (Figure 6Aii), it is unclear if this difference is large enough to explain the difference in the contribution to the SEP. The difference in the contribution of L5-L5 excitation to the SEP could potentially be attributed to changes in membrane excitability due to rewiring of inhibitory connections.

The difference between the SM and the original circuit can be largely attributed to the presynaptic contribution of layer 5 large basket cells and layer 5 nest basket cells (collectively called perisomatic targeting cells [PeriTCs]) to layer 5 pyramidal cells (Figure 6B). We note, however, that while the difference in the PeriTC contributions between the SM and original circuits is a substantial contributor to the total difference, the contributions of populations, such as thalamic inputs to L5, and inhibitory inputs from other layers to L5 and L6 are also substantial (Figure 5E), but partially cancel out.

For PeriTCs, synapse locations are closer to the soma in the SM circuit. The firing rates of PeriTCs do not differ substantially between the original and the SM circuits (Figure 6Ci). Moreover, the firing rates of PeriTCs do not differ substantially between the SM circuit and a circuit with disconnected cortico-cortical connectivity that receives only thalamocortical input (Figure 6Cii), implying that the activity of these cells is driven by direct thalamic input (as expected, given that inhibitory cells respond to thalamic stimulus with low latency^17^). This indicates that the difference in their contributions must be attributable to the difference in their synapse locations, which demonstrates that changes in local connectivity can have relevant impacts on EEG signals, without significantly affecting their firing rates. This confirms the findings of Rimehaug et al., 2023,^19^ of a similar dissociation between firing rates and electrical signal, but for the CSD signal instead of the EEG.

In the SM circuit, PeriTC synapses are moved from apical compartments to perisomatic compartments (Figure 6F). For a random sample of L5PCs in our model, we visualize the sensitivity of the EEG to transmembrane currents in each compartment (see Tharayil et al.^8^ for details). Note that because of the gauge degree of freedom of the electric potential, these “compartment weights” are only defined up to a constant offset, i.e., only the gradient in weights over a neuron affects the signal. We, therefore, shift the compartment weights such that the range of compartment weights over the population is centered on zero. We observe that the perisomatic compartments have more negative weights than the apical compartments (Figure 6E). With synapses primarily targeting perisomatic regions, the neuron acts as a dipole, with currents at the soma and associated return currents along the apical dendrite. Because of the large gradient in weights between the soma and apical dendrites, concentrated inhibitory synaptic input near the soma would lead to a more negative deflection in the EEG than the same input distributed over the dendritic arbor.

### Specificity of layer 5 inhibition drives differences in width of the N1 component *in silico*

**Figure 7:**
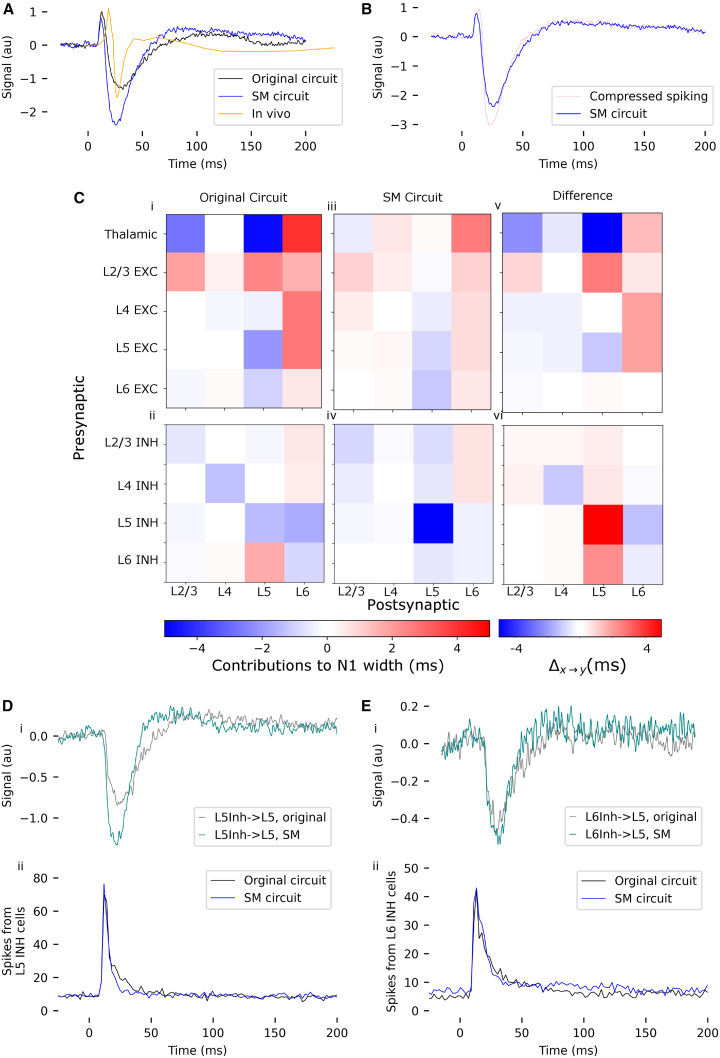
Investigation of N1 component width (A) Comparison of SEP signals from the original and the SM circuit with *in vivo* data obtained on postnatal day 16.^3^ (B) Compressing the timing of spikes from layer 5 PeriTC neurons does not have a substantial impact on the SEP. (C) (i and ii) $FWHM_{o,x\to y}$: Contribution of various presynaptic-postsynaptic pathways to the width of the N1 component in the original circuit. A positive contribution implies that the pathway widens the N1 component. (iii and iv) $FWHM_{sm,x\to y}$: The same, but for the SM circuit. (v and vi) $\Delta_{x\to y}$: Contributions of different presynaptic-postsynaptic pathways to the difference in the width of the N1 component between the original and the SM circuit. A positive contribution implies that the pathway narrows the N1 in the SM circuit, relative to the original circuit. (D) (i) Contribution of L5-L5 inhibition to the SEP, for both the original and SM circuits. (ii) Firing rate of L5 inhibitory cells in the original and SM circuits. (E) Same as (D), but for L6 inhibitory cells.

As previously described, the width of the N1 component *in silico* is approximately 3 times longer than that of the *in vivo* signal (Figure 7A). The SM circuit produces an N1 component 25% narrower than the original, with a full width at half maximum (FWHM) of 21 and 28 ms, respectively.

In order to determine whether more precise spike timing in the PeriTC cells would bring the SEP from the SM circuit closer to *in vivo* observations, we replay the spikes from the fully connected circuit into a disconnected circuit, with all PeriTC spikes occurring between 10 ms post-stimulus and 40 ms post-stimulus shifted to 15 ms post-stimulus (i.e., the peak of the spike histogram [Figure 6Ci]). Even though this level of compression is highly exaggerated, the resulting SEP is not substantially different from the SEP in the SM circuit—the amplitude of the N1 component is slightly greater, and the time course of the N1 is slightly faster (Figure 7B).

To determine why the width of the N1 component is so much longer than *in vivo*, we calculate the contribution of each presynaptic-postsynaptic pathway to the FWHM of the N1. We denote the width of the N1 component in the original circuit *FWHM*_*o*_ and that in the SM circuit *FWHM*_*sm*_. We calculate, for each combination of presynaptic population *x* and postsynaptic population *y*, the SEP signal with the contribution of that particular presynaptic-postsynaptic pathway removed, in both the original and the SM circuits. We denote the resulting signals $S_{o,x\to y}$ and $S_{sm,x\to y}$ for the original and the SM circuits, respectively. The width of the N1 component in each of these signals is $FWHM_{o,x\to y}$ and $FWHM_{sm,x\to y}$. The quantities $FWHM_{o}-FWHM_{o,x\to y}$ and $FWHM_{sm}-FWHM_{sm,x\to y}$ represent the contribution of the pathway *x*→*y* to the FWHM of the SEP in the original and the SM circuits, respectively. A positive value implies that the pathway lengthens the N1 component, while a negative value implies that it shortens the N1 component.

We find that in both the original and the SM circuit, the thalamus-L6 pathway lengthens the N1 component (Figures 7Ci and 7Ciii). In the original circuit, recurrent excitatory input to L6 also lengthens the N1 component (Figure 5Ci), although this effect is smaller for the SM circuit (Figure 5Ciii).

We denote the difference in the width of the N1 component between the original and SM circuits as *FWHM*_*o*_−*FWHM*_*sm*_ = Δ*FWHM*. The extent to which a particular presynaptic-postsynaptic pathway contributes to the difference Δ*FWHM* in the width of the N1 component in the SM circuit relative to the original circuit can be inferred by the effect of removing the pathway. Thus, for each pathway, we calculate $\Delta_{x\to y}=\left(\Delta FWHM-\Delta FWHM_{x\to y}\right)$. A positive value of $\Delta_{x\to y}$ implies that removing the pathway *x*→*y* reduces the difference Δ*FWHM* between the N1 component of the original and SM circuits and therefore, that the pathway *x*→*y* could contribute to the observed difference.

We find that in our models, L5-L5 inhibition contributes strongly to Δ*FWHM* (Figure 7Bvi), as does, to a lesser extent, L6-L5 excitation (Figure 7C.v), among other populations. The time constant of recovery of the contribution of L5-L5 inhibition to the SEP is faster in the SM circuit than in the original (Figure 7Di). Due to the impact of noise on the signal when removing populations (see section sum of estimated presynaptic contributions approximates total signal), we were unable to attribute the narrowing of the N1 component to a more specific population. However, as firing rates for layer 5 excitatory cells are not substantially different between the original and SM circuits (Figure 7Dii), the differences in the time course of the SEP contribution must be due to differences in synapse placement in the SM circuit. Unlike the contribution of L5-L5 inhibition to the SEP, the contributions of L6-L5 inhibition do not differ substantially between the original and SM circuits (Figures 7Ei and 7Fi). These populations contribute to the increased width of N1 in the original circuit only because they are a relatively larger share of the N1 component in the original circuit than in the SM circuit.

## Discussion

We have simulated the SEP induced by a whisker flick in two *in silico* models of the rat non-barrel primary somatosensory cortex. In both cases, the resulting SEP has the basic shape observed *in vivo*, with an initial positive deflection (P1) followed by a negative deflection of similar amplitude (N1). While the origins of the SEP waveform cannot be fully explained, we were able, through careful simulation techniques, to decipher the contributions of individual neuronal populations in our model to the characteristics of the two components. We further simulated the LFP and CSD from our model and found that the CSD largely recapitulated the features of the *in vivo* CSD, albeit with the addition of a current sink in L5 that did not appear in the *in vivo* data.

Our main findings (cf. Table 2) are that, in our model.

• The P1 component is driven primarily by thalamic input to L2/3 and L5.
• The N1 component is driven in part by thalamic input to L6 and recurrent L5-L5 inhibition.
• Increased perisomatic inhibition increases the amplitude and reduces the width of N1.

**Table 2:** Comparison of P1 and N1 features across different circuit configurations

	P1 amplitude	N1 amplitude	N1 width
In the original circuit	Is dominated by thalamus-L2/3 and thalamus-L5 connections	Is driven by connections from thalamus to L6, thalamus to L5, and L5INH to L5	Is dominated by the thalamus-L6 and L5INH-L5 connections
In the SM circuit	Is the same as in the original circuit	Is larger than in the original circuit stronger, due to greater impact of the L5-INH connection circuit	Is shorter than in the original circuit, due to faster L5INH-L5 response
If TC synapses exclusively targeted L4	Would likely be reduced, as the added contributions from thalamus-L4, and L4 to L2/3 and L5, would be less than the reduction in the contribution from thalamus to L2/3 and L5	Would be reduced, due to the elimination of thalamus to L6 and thalamus to L5 connections	Would be much faster due to elimination of thalamus-L6 contribution
If TC synapses formed preferentially on basal dendrites	Would be increased, because thalamic synapses onto apical dendrites of L6 PCs, which produce a negative contribution, would be moved to basal dendrites of L5 PCs, where they would produce a positive contribution. Similarly, apical synapses on L5 PCs would become basal synapses on L2/3 PCs	Would be reduced, due to the elimination of the thalamus to L6 connections	Would be much faster due to elimination of thalamus-L6 contribution
If S1 received feedback from higher-order cortical regions	Would likely not change	Would likely increase	Would be faster, possibly due to inhibitory input to L5

We note that the predictions we make about the SEP in a configuration where TC synapses formed preferentially on basal dendrites equally apply to a preference for thalamocortical synapse formation on layer 4 neurons.

Interestingly, these changes in the N1 component are not explained by changes in pre-synaptic spiking but in how the synaptic currents associated with the spiking affect the signal. A dissociation between spiking and the CSD signal has been previously described.^19^ Our work thus extends this observation of a dissociation between firing rates and electrical signals to the EEG.

While our model does replicate some of the key features of the SEP, there are some important differences in both the shape of the signal and its amplitude. These differences may in part be the result of differences in connectivity between the model and *in vivo* circuits; future work may modify these parameters in the model in order to optimize the *in silico* EEG to match *in vivo* data. These possibilities are discussed in further detail in the section differences between in silico and in vivo signals and Table 2.

### SEP reflects thalamocortical excitation and recurrent inhibition

We have shown that the main features of the SEP, namely, the N1 and the P1 components, can, in principle, be generated entirely by direct thalamocortical input, with the N1 component being driven by thalamic input to L6 neurons (Figures 5Av and 5Cv). In our model, the P1 component is primarily driven by direct thalamic input to L2/3 and L5, but it is, to a certain extent, modulated by L5-L5 inhibition (Figures 5Biv and 5Div and Table 2). While in our models thalamic input to L6 contributes to the N1 component—particularly to its late phase—recurrent connectivity has a larger impact on N1 than thalamic innervation. In particular, cortico-cortical inhibition has a substantial impact on the amplitude of the N1 component (Figures 5Bi and 5Di and Table 2), although in the original circuit, this is balanced by the effect of cortico-cortical excitation (Figure 5Ai).

*In vivo*, the N1 component is present from at least P7. Its width peaks on P10, before narrowing as the animal ages; after P16, its amplitude begins to decrease slightly.^3^ In our model, the ratio of the amplitude of the P1 and N1 components is similar to that on P16 in the original circuit, and between that of P13 and P16 in the SM circuit. The N1 component has a width closest to, but somewhat larger than, the width of the *in vivo* signal on P10.

The width of the S1 component in the SM circuit more closely resembles that of the *in vivo* signal than that of the original circuit. We have shown that this is largely due to the impact of L5-L5 inhibition. It has been found that activation of distal synapses can lead to longer-lasting depolarization^20^; the reduction in N1 width in our SM circuit may, therefore, be attributable to the more specific somatic targeting of inhibition.

Our results contrast sharply with those of Bruyns-Haylett et al.,^2^ who found that the application of a GABA antagonist had no effect on P1 or the initiation of N1, but increased the width of the N1. In contrast, we find that in our models inhibition plays a significant role in the initiation of N1, while the width of N1 is determined primarily by thalamic input to layer 6 (Figure 5). It is important to note, however, that the experiments in Bruyns-Haylett et al.^2^ used adult animals and, moreover, the intervention in that study was applied in a “closed-loop” manner, i.e., the application of the GABA antagonist could have affected the spiking activity of local circuits, which was explicitly avoided in our approach. While Bruyns-Haylett et al.^2^ found no significant differences in resting-state multi-unit activity after the application of the GABA antagonist, it is unclear what effects it had on evoked activity. Given the complexity of the contributions to the N1 component in our model, it is difficult to predict how a GABA antagonist would affect the EEG in a closed-loop format; future work that explicitly models the GABA antagonist in a closed-loop configuration may help explain the findings in Bruyns-Haylett et al.^2^

### Implications for development

*In vivo*, the P1 component of the whisker flick EEG emerges around P13 and increases in amplitude over the course of development (Figure 2C)^3^. Our finding that the P1 component in our model is driven primarily by thalamic input suggests that the emergence of the P1 component over the course of development is not necessarily related to changes in local connectivity, although decreases in L5-L5 inhibition during maturation may partially account for the observed increase in the amplitude of the P1 component. Rather, we speculate that the increased P1 component may result from increased thalamic input to L2/3 and L5, or morphological changes in L2/3 and L5 pyramidal cells. It has previously been shown that the strength of thalamic innervation of L5 pyramidal cells does increase over the course of development^21^ (albeit in mouse rather than rat). Further *in silico* and *in vivo* experiments could test these hypotheses.

The earlier timing of the P1 in our model might be attributed to age-related differences in the timing of thalamic spikes in our model compared to *in vivo*. While spike times in our model are based on an *in vivo* PSTH,^7^ these data were obtained from adult animals, while the *in vivo* SEPs were obtained from juveniles. As the onset of the SEP occurs earlier as the animal matures (Quairiaux et al.^3^; cf Figure 2C), it seems likely that this explains the discrepancy in the timing of the SEP. In our model, axonal delays for the transmission of thalamic action potentials are calculated based on the distance from the bottom of layer 6 to the synapse location, ignoring the length of the axon in the thalamus itself; this may also contribute to the faster latency in our model.

That the N1 component of the SEP is so strongly affected by recurrent inhibition suggests that maturation of inhibitory circuit connectivity, particularly within L5, may be responsible for the reduction in N1 amplitude observed over development (Figure 2C; Quairiaux et al.^3^), although as previously discussed, inputs from outside the somatosensory cortex are also likely to contribute to the N1. Further research is needed in order to fully explain the changes in the SEP over the course of development. It is almost certain that changes in cellular anatomy and physiology during the course of development also impact the SEP. To a limited extent, the influence of changes in anatomy on the SEP can be approximated by scaling the weighting factors for each neural compartment to mimic the effects of a spatial rescaling of the neuron. However, it may be necessary to generate new morphologies and reoptimize neuron electrical models to fully capture the cellular-level changes that occur during development.

### Differences between *in silico* and *in vivo* signals

While our model replicates the key features of the SEP, there are important differences between our model’s outputs and *in vivo* data. These can be broadly divided into differences in the shape of the signal and amplitude differences. While our model produces smaller EEG amplitudes than reported in the literature (Figure 4), it is not unexpected that the model fails to replicate *in vivo* SEP amplitudes for a specific paper: there is more than an order of magnitude variation in the literature-reported *in vivo* SEP peak-to-peak amplitudes, which ranges from 40 *μm*V^13^^,^^14^^,^^15^ to 750 *μm*V.^3^ Reported LFP magnitudes also vary substantially, with Riera et al.^13^ reporting three times lower peak LFP amplitudes than typical literature values.^3^^,^^16^^,^^17^ Thus, any model cannot be consistent with the entirety of literature. We confirmed that the here-calculated EEG magnitudes are consistent with the computed LFP magnitudes (section model EEG and LFP amplitudes are self-consistent). A potential explanation for smaller modeled SEP magnitudes is that we only simulate a subvolume of the somatosensory cortex and might not capture the full extent of neural activation due to whisker flick. In addition, our model simulates the deflection of a single whisker, while all whiskers on one side are stimulated in Quairiaux et al.^3^ As the model can readily be expanded to incorporate the entire somatosensory cortex, and stimulus parameters could be adapted to simulate more extensive whisker flick input, the ability of such changes to account for variation in SEP amplitudes *in vivo* could be studied in future work.

Due to the lack of amplitude agreement with the *in vivo* SEP, we concentrate on comparing signal shapes by normalizing the amplitude of the *in vivo* P1 to that of the *in silico* signal from the original circuit. Because this normalization also reveals a near-perfect alignment between the P1 peak in the *in vivo* signal and that of the SM circuit, we can compare the shapes of all three signals. The relative amplitude of the N1 component is much larger in the SM circuit than either *in vivo* or in the original circuit. We, therefore, conclude that the original circuit provides a better fit to the *in vivo* data than the SM circuit, although it is not possible to confirm whether this is due to an overly large N1 component in the SM circuit or due to a too-small P1 component.

We were surprised by the fact that the incorporation of connectivity trends from the MiCrONS dataset worsened the agreement in the relative amplitudes of the P1 and N1 components between our model and the *in vivo* data. The *in vivo* SEPs presented in this paper were taken from an animal on P16, and the *in silico* model was built using data from a variety of sources with varying ages. The MiCrONS dataset is, in contrast, obtained from a mature animal.^12^ It may be that the connectivity trends observed there emerge later in development. Because maturation is associated with a reduction, rather than increase, in the amplitude and width of the N1 component,^3^ this alone does not account for our observations. It may be that our *in silico* model also excludes other changes in anatomy and physiology over the course of maturation, which compensate for the deepening of N1 by increased specificity of PeriTC targeting.

Key differences in the shape of the EEG between our models and *in vivo* data include an earlier P1 component and a wider N1 component *in silico*. These differences may be attributable to differences in the connectivity of the circuit *in silico* compared to *in vivo*. Identifying changes in the connectivity of the *in silico* circuit that produce more realistic EEG signals might, therefore, be a useful method to develop hypotheses about *in vivo* circuit connectivity. Here, we consider three potential changes to our *in silico* connectivity that may improve the fit between the *in silico* and *in vivo* EEG.

• The use of the “canonical” model of thalamocortical organization.
• Implementation of a preference for thalamocortical synapse formation on basal synapses.
• Addition of feedback input from higher-order cortical regions.

#### Canonical model of circuit organization

Our model is not based on the canonical model of cortical organization, in which thalamic inputs project primarily to layer 4, which then distributes this input to L2/3 and L5,^22^ with slower and weaker connections to L5 than to L2/3. Instead, thalamic fibers in our model project primarily to layer 5 (Figure 1D), as well as to L2/3 and L4, because it is built using a bottom-up approach, where thalamocortical synapses are placed in order to match known Bouton densities, without preferentially targeting specific cell types.^5^^,^^6^ *In vivo* data have shown that layers other than L4 do receive direct thalamocortical input^23^; moreover, thalamic input exclusively targeting layer 4 would be inconsistent with the experimentally-observed existence of a peak in thalamocortical Bouton density at the bottom of L5.^5^ A strict adherence to the canonical model, with no direct thalamic innervation to any layers besides L4, would therefore be inconsistent with the available data. Given that our model accurately reproduces circuit physiology,^6^ as well as some of the key features of the SEP and CSD (Figures 2D and 2E), it is plausible that thalamocortical innervation is not organized as strictly as the canonical model would suggest.

However, several lines of evidence suggest that our model may underestimate the specificity of thalamocortical targeting of L4. *In vivo*, MUA tends to begin in L4 before spreading to other layers,^3^^,^^16^ but in our model, spiking is initiated in L6 and spreads upward, in line with the relative axonal delays of our thalamocortical innervation (Figure 1E).^6^ Moreover, our model has a large CSD sink at the bottom of L5, which is not reflected in the *in vivo* data (Figure 2E). This sink is driven by L5 and L6 pyramidal cells (Figure S3), occurs immediately after stimulation, and is aligned with a peak of thalamocortical Bouton density, suggesting that it is evoked by thalamocortical currents impinging on L5 and L6 PCs. Einvoll et al.^16^ have argued, based on the observation that the MUA precedes the LFP *in vivo*, that thalamic input does not directly evoke LFP signals; rather, thalamic input drives spiking in L4, which in turn evokes LFP in L2/3 and L5, localized primarily in the upper part of the cortical column. A thalamic innervation profile, which more closely resembled the canonical circuit model, might, therefore, result in a more realistic CSD signal.

If our circuit more closely followed the canonical model, we would expect the P1 component to be driven not by direct thalamic innervation of L2/3 and L5 but by disynaptic innervation mediated by L4, which would result in a later, and, therefore, more accurate timing of the P1 peak compared to *in vivo* data (Table 2). The removal of thalamic synapses on L6 pyramidal cells would also result in a significant reduction in the amplitude and width of the N1 component (Table 2).

Previous work using *in silico* circuits based on the canonical model has simulated the magnetoencephalogram (MEG) produced by tactile input in nonhuman primates^24^ and in humans by median nerve stimulation.^25^ Our work confirms the finding in these papers that thalamocortical input to L2/3 pyramidal neurons generates an initial positive deflection. (Note, however, that in the human/nonhuman primate literature, the initial positive deflection is typically labeled N1, and the subsequent negative deflection P1, i.e., the opposite of the convention used in this work. For clarity, we will hereafter use the terms “the initial positive deflection” and “the subsequent negative deflection” to refer to, respectively, P1 and N1 as defined in this paper, when comparing primate and rodent studies).

As in our work, both Jones et al.^24^ and Thorpe et al.^25^ find that perisomatic inhibition is largely responsible for the subsequent negative deflection in the electrical signal. As our model is of rat cortex and Jones et al.^24^ and Thorpe et al.^25^ model primate cortex, this observation may reflect a circuit motif that is conserved across species.

However, the findings in Jones et al.^24^ and Thorpe et al.^25^ differ in some respects from ours. While we see a significant contribution to the initial positive component due to thalamocortical input to L5 pyramidal cells, Thorpe et al.^25^ find no positive deflection from L5 PCs, while in Jones et al.,^24^ this positive deflection is delayed, so that it occurs during the negative deflection of the total signal. In addition, Thorpe et al.^25^ report a larger negative component in postsynaptic L2/3 than we do.

The use of the canonical model in Jones et al.^24^ and Thorpe et al.^25^ provides a direct explanation for the lack of a large positive contribution of thalamic afferents to L5; it may also be the case that the canonical model’s stronger input to L2/3 results in greater evoked activity in L2/3 interneurons, leading to the larger negative contribution from L2/3 in Thorpe et al.^25^ We would, therefore, expect a more “canonical” organization in our *in silico* circuits to result in a reduction in the relative amplitude of the initial positive component (Table 2).

#### Preference for synapse formation on basal dendrites

Alternatively, it may be the case that the differences between *in vivo* and *in silico* results are due to the neurite types onto which thalamocortical synapses are placed. In our model, thalamocortical innervation of L5 PCs has a bimodal distribution, with one peak targeting apical dendrites and the other targeting basal dendrites. However, previous studies have found a unimodal distribution of thalamic synapses onto L5 pyramidal cells, primarily targeting basal dendrites.^26^ Therefore, it may be that thalamocortical projections preferentially target basal dendrites, in which case the synapses on apical dendrites of L5 PCs should be moved to L4 neurons.

Speculatively, if a preference for basal innervation holds for all cell types, we would expect a reduction in the amplitude and width of the negative component, due to the transfer of thalamocortical synapses from apical dendrites of L6 PCs to basal dendrites of L5 PCs (Table 2). As thalamic input to L5 also produces a slow negative contribution to late negative (Figures 7Ci and 7Ciii), this would not entirely account for the increased duration of the negative in our model. However, the negative deflection due to the thalamus-L5 pathway is likely due to synapses on the apical dendrites of L5 PCs, which would also be removed by the basal targeting preference. Thus, we expect that such a preference would significantly shorten the negative component (cf. Table 2). The addition of a preference for basal synapses may also increase the amplitude of the P1 component of the LFP and the EEG; the elimination of apical synapses on L2/3 and L6 pyramidal cells would result in a more spatially concentrated current source distribution (by removing the superficial current sink associated with apical synapses on L2/3 PCs and the deep current source associated with return currents in L6 PCs), possibly leading to larger-amplitude LFPs and more aligned current dipoles, resulting in larger EEG amplitudes (Table 2).

#### Feedback input from higher-order regions

Another alternative is that our model excludes feedback from higher-order regions, which influences the EEG signal. It has been suggested that, beginning around 25 ms post-stimulus, activity in the contralateral hemisphere has a significant effect on the recorded SEP^27^; this aligns with the time course of the termination of the negative component *in vivo*. Activation of the contralateral hemisphere increases over the course of development, correlating with a reduction in the width of the negative component.^3^ Given that our model produces a negative component with width close to that of the *in vivo* negative component on P10, before the contralateral hemisphere begins to be activated, it is possible that inter-hemispheric activity, which is not represented in our model, is responsible for the reduction in the width of the N1 (cf. Table 2). The lack of contralateral input may also explain the relatively low amplitude of the CSD in our model after ∼25 ms.

#### Improving the fit between *in silico* models and *in vivo* data

In this paper, we use an exemplary modification—the implementation of perisomatic targeting from the SM connectome—to demonstrate that small changes in the connectivity of the circuit can have significant impacts on the EEG without significantly changing firing rates (Figure 6). It thus lays the foundation for the use of EEG in constraining circuit models, which we hope will be explicitly demonstrated in the future. The use of simulated electrical signals, in the form of CSD and MEG, has previously been proposed as a metric to constrain model building.^19^^,^^25^ Thorpe et al.^25^ suggested that input to supragranular layers, possibly from higher-order cortical regions, is necessary for the generation of the secondary negative component in the human SEP induced by median nerve stimulation (denoted P1 in the human literature, but corresponding to N1 according to our terminology). Similarly, Rimehaug et al.^19^ found that altering synapse placement and adding feedback connections from higher-order areas could improve the fit between *in vivo* and *in silico* CSD, demonstrating the validity of using electrical signals to constrain neural models. Indeed, validating the CSDs produced by our model, may be useful in constraining modifications made to replicate EEG in different states and stages of development.

However, we believe that modifying circuit parameters to optimize the fit between *in silico* and *in vivo* EEG is out of scope for this paper. In this paper, we lay a foundation for future work by demonstrating the calculation of EEG signals from detailed neural circuit models and explaining the biophysical bases of these signals. We hope that future work will expand on the results presented here by introducing refinements to the circuit model that are predicated on biological insight, such as those proposed above. These manipulations may indeed provide novel biological insight into the structure of the somatosensory microcircuit.

### Limitations of the study

Our approach to deciphering the contributions of populations relies on the assumption that the contributions of pre-synaptic populations sum up approximately linearly. This assumption has been used and validated before for the CSD signal.^9^ Additionally, we validate that presynaptic contributions calculated using our method do sum approximately to the total EEG signal produced by our models (Figure 3), indicating that our modifications likely do not place the circuit in an entirely different dynamical regime. However, we also found that rewiring inhibitory connectivity modulated how excitatory pre-synaptic populations contributed to the signal (Figure 6Ai), even though their spiking activity remained unchanged (Figure 6Aii). This demonstrates complex, non-linear interactions between populations and the limitations of the assumption. While this assumption may be approximately true for a given circuit, it must be re-evaluated *globally* even for small *local* changes to a model.

A potential limitation of this study is the application of whisker flick stimulus to non-barrel somatosensory cortex; it is not clear that nbS1 would produce the same responses to thalamic input as barrel cortex. However, this is not a fatal limitation, as the thalamic input is modeled on innervation of the barrel cortex^5^ and the model has been shown to replicate neural activity in the barrel cortex in response to whisker flick and optogenetic manipulation.^6^ It has been shown to reproduce both the population-level magnitude and the time course of spiking responses to these stimuli.

While our *in silico* models are extensively validated, there remain aspects that may differ from *in vivo* conditions in ways that might influence the calculated SEP. The simulated synaptic physiologies have been shown to produce excitatory postsynaptic potentials with *in vivo*-like amplitude and time course, but these data have been recorded at the soma, rather than in the dendrites. Similarly, the electrical models of the neurons have been validated on the basis of responses to stimuli recorded at the soma.^6^ Particularly for pyramidal cells, with their extended dendritic arbors, the contribution to the EEG is strongly influenced by transmembrane currents in the dendrites as well as the soma. Thus, differences in the electrophysiological properties of the dendrites may influence the simulated SEP.

We observed that relatively small alterations to local connectivity may significantly impact the simulated SEP. While the large-scale connectivity trends in our circuit model are well validated,^5^ local connectivity may benefit from the incorporation of more data obtained from electron microscopy or other methods. In particular, it may be necessary to incorporate connectivity data obtained at specific points during maturation.

We assume that thalamic neurons do not have a significant presynaptic contribution to the EEG signal. This assumption is justified on the basis that the EEG signal is driven primarily by synaptic currents^28^; as only the axon terminals of the thalamic fibers are located in the cortex, there is no current input to the thalamic fibers sufficiently close to the recording electrodes to be measurable. However, recent research has suggested that action potentials may also measurably contribute to the EEG.^29^ As the thalamic fibers are modeled as virtual inputs to the cortical neurons rather than in biophysical detail, the currents generated by action potentials in the thalamic fibers are ignored.

## Resource availability

### Lead contact

Requests for further information should be directed to Joseph Tharayil (tharayiljoe@gmail.com).

### Materials availability

This computational study used no materials.

### Data and code availability

The simulations performed in this study are run using the Neurodamus^30^ simulation control tool, which integrates with the CORENEURON^31^ computation engine. To calculate EEG signals, Neurodamus relies on a “weights file,” which lists the sensitivities of the EEG to the currents from each neural compartment in the model. Weights files for the calculation of EEG signals are created using BlueRecording.^8^

• Code for running the simulations and generating the figures in this paper (excluding Figure 2E) are available at https://github.com/joseph-tharayil/whiskerFlick. Postprocessed EEG traces from these simulations are available on Zenodo under the following doi: https://doi.org/10.5281/zenodo.14442089.
• Code for generating Figure 2E is available at https://github.com/joseph-tharayil/csd_paper/tree/whiskerFlick_paper. Postprocessed LFP traces used to generate this figure are available on Zenodo under the following doi: https://doi.org/10.5281/zenodo.14998743.
• The source code for BlueRecording is available at https://github.com/BlueBrain/BlueRecording.
• The seven-column subvolume of the BBP circuit model is available on Zenodo under the following doi: https://doi.org/10.5281/zenodo.11113043. The SM circuit is available under the following doi: https://doi.org/10.5281/zenodo.11108303.
• Membrane mechanism files used in the neuro-simulations are available at https://github.com/BlueBrain/neurodamus-models.
• Neurodamus is available at https://github.com/BlueBrain/neurodamus.
• CoreNEURON itself is fully integrated into the NEURON simulation environment, which is available at https://github.com/neuronsimulator/nrn.
• Code for the generation of FEM models, as well as a list of dependencies, are available at https://github.com/BlueBrain/BlueBrainHeadModels. The required finite element meshes are available on Zenodo (https://doi.org/10.5281/zenodo.10926947).
• Finite element meshes used in the simulations are available on Zenodo (https://doi.org/10.5281/zenodo.14419388) and oSparc (https://osparc.io/#/study/2d25d5be-b667-11ef-baa3-0242ac177740).

## Acknowledgments

We thank Kevin Eason and Mayuko Sasaki-Kuroiwa for graphic design support. This work was supported by funding to the Blue Brain Project, a research center of the 10.13039/501100001703École Polytechnique Fédérale de Lausanne, from the Swiss government’s ETH Board of the Swiss Federal Institutes of Technology.

## Author contributions

Conceptualization, J.T., E.N., and M.R.; methodology, J.T. and M.R.; software, J.T.; validation, J.B.I.; formal analysis, J.T. and J.B.I.; investigation, J.T.; resources, E.N. and M.R.; writing – original draft, J.T.; writing – review and editing, J.T., J.B.I., E.N., and M.R.; visualization, J.T.; supervision, E.N. and M.R.

## Declaration of interests

The authors declare no competing interests.

## STAR★Methods

### Key resources table

**Table:** 

REAGENT or RESOURCE	SOURCE	IDENTIFIER
**Deposited data**		

Postprocessed EEG traces	Zenodo	https://doi.org/10.5281/zenodo.14442089
Postprocessed LFP traces	Zenodo	https://doi.org/10.5281/zenodo.14998743

**Software and algorithms**		

BBP nbS1 original circuit	Zenodo	https://doi.org/10.5281/zenodo.11113043
BBP nbS1 SM circuit	Zenodo	https://doi.org/10.5281/zenodo.11108303
Neurodamus	Github	https://github.com/BlueBrain/neurodamus
Neuron	Github	https://github.com/neuronsimulator/nrn
Finite element meshes	Zenodo	https://doi.org/10.5281/zenodo.14419388
BlueRecording	Github	https://github.com/BlueBrain/BlueRecording
Simulation and analysis code for EEG	Zenodo	https://github.com/joseph-tharayil/whiskerFlickhttps://doi.org/10.5281/zenodo.17481979
Simulation and analysis code for CSD	Zenodo	https://github.com/joseph-tharayil/csd_paper/tree/whiskerFlick_paperhttps://doi.org/10.5281/zenodo.17484485

### Method details

#### Neural circuit models

We simulate the central, 7-column subvolume of the BBP model of the nbS1.^6^ This subvolume contains ∼210,000 biophysically-detailed neurons, which receive Ornstein-Uhlenbeck conductance noise from virtual sources, to represent the effect of synapses from non-modelled regions. Noise input is optimized to produce *in silico* firing rates equal to desired ratios (*P*_*FR*_) of *in vivo* firing rates, corresponding to different levels of excitability (in this paper, *P*_*FR*_ = 0.3), with the ratio of the standard deviation of the noise to the mean of the noise fixed at 0.4 (*R*_*OU*_ = 0.4).^6^ Simulations are conducted with a simulated extracellular calcium concentration (which modulates the efficacy of synaptic transmission) of 1.05 mM. The subvolume is innervated by virtual thalamic fibers representing VPM inputs; a virtual whisker flick stimulus activates 10% of VPM fibers, with randomly-sampled spike times obtained from an *in vivo* PSTH of VPM activity in response to whisker deflection, as in^6^. The delay between thalamic spike times and synapse activation (accounting for axonal transmission time) is based on the distance from the bottom of Layer 6 to the synapse location.

In addition to the original subvolume, we also study a rewired circuit,^11^ in which the targeting of interneurons is modified to more closely match the MiCrONS dataset.^5^^,^^12^ We refer to this circuit as the Schneider-Mizell (SM) circuit. Parvalbumin-positive (PV+) interneurons target perisomatic regions with more specificity, vasoactive intestinal peptide-expressing (VIP+) interneurons preferentially target inhibitory neurons, and Layer 1 and neurogliaform interneurons have primarily monosynaptic connections. Synapses and input noise parameters are recalibrated for the SM circuit, in order to ensure that firing rates match *in vivo* data.^6^

#### Calculating EEG signals

We calculate the EEG signal from our neural circuit models using the “reciprocity approach”^32^: The contribution to the EEG of a transmembrane current in a particular neural compartment is the product of the current magnitude and the electric potential that would be produced at the location of the compartment by a unit current applied between the recording and the reference electrode. The workflow for calculating EEG signals using BlueRecording is described in detail in^8^ and briefly summarized below.

(1) We created a finite element model of the rat head, with a recording electrode directly over the forelimb region of the somatosensory cortex and a reference electrode over the hindlimb region. Using Sim4Life (Zurich MedTech AG, Zurich, CH), we calculated the electric potential in the head generated by a current applied between the electrodes.
(2) Using BlueRecording, we interpolated the potential field at the location of each neural compartment in the model and wrote the values to a “weights file”.
(3) At runtime, the Neurodamus simulation^30^ control program loads the weights file and, at each time step, calculates the EEG as the dot product of the transmembrane currents and the weights. At the end of the simulation, an HDF5 file is produced, which reports the contribution to the EEG for each neuron.
(4) In postprocessing, we summed the contributions of all neurons from populations of interest.

#### Calculating LFP and CSD signals

We calculate the LFP recorded in our original circuit model using a linear electrode array, located in the center of the circuit and oriented along its vertical axis. Electrodes are spaced 80 *μm* m apart. We calculate LFP signals from our model using the line-source approximation.^33^ On the grounds that the LFP electrodes and the volume of neural tissue recorded is small relative to the entire brain, the line source approach assumes that neural segments can be treated as 1-dimensional lines of current (with somas treated as 0-dimensional points) inside an infinite medium with homogeneous and isotropic conductivity. As for the EEG signals, LFP signals are calculated using BlueRecording; however, segment coefficients are calculated analytically rather than by using the finite element approach.^8^ From the recorded LFP, the current source density (CSD) is calculated using the standard CSD method with Vankin correction,^34^ as implemented by Rimehaug et al.^19^

To estimate current dipoles from our LFP signal, we first estimate the CSD using the step-iCSD method.^35^ This method assumes that the current source density is homogeneous within a cylindrical disk centered on the electrode and perpendicular to the electrode array, with height *h* equal to the inter-electrode spacing, and radius *ρ* which we set to 500 *μm*, based on the assumption that the LFP reflects neural activity within one column and half of each of the neighboring columns.^16^ To estimate the peak dipole moment density *p* in our model (with respect to cortical surface area), we then calculate the integral $p=\int_{0}^{z_{\max}}z\cdot CSD\left(z,t\right)dz$, where z is the depth along the electrode array and t is the time of the peak current density. Using the reciprocity theorem,^32^ we then estimate the peak EEG signal from a patch of cortical tissue with radius *r* as $\pi r^{2}*p*\hat{E}$, where $\hat{E}$ is the average dipole-projected lead field (i.e., the component parallel to the CSD-dipole of the E field produced by a unit current applied between the recording electrodes) over the patch of tissue.

#### SEP simulations

##### Unmodified circuits

For both the original and the SM circuit, we simulate 10 trials, each of which has a different random seed for the conductance noise and the selection of thalamic fibers. We refer to this set of trials as a *simulation campaign*. In each trial, two thalamic stimuli are simulated. The EEG signal is calculated as described above; in postprocessing, we isolate the SEP produced by the second whisker flick stimulus, in order to account for the effects of accommodation. We apply a second-order bandpass Butterworth filter with cutoff frequencies of 1 Hz and 500 Hz. The SEP is averaged over the 10 trials. Signals from both the original and SM circuits are normalized to the peak amplitude of the P1 component in the *in silico* EEG from the original circuit.

##### Isolating postsynaptic contributions

BlueRecording outputs the contribution to the EEG signal from each neuron individually. Because of the linearity of Maxwell’s equations, we can isolate the contribution of a particular postsynaptic population by summing the contributions of all neurons belonging to that population. In this paper, we isolate the postsynaptic contributions of inhibitory neurons in Layer 1, and both excitatory and inhibitory neurons in layers 2/3, 4, 5, and 6.

##### Isolating presynaptic contributions

To isolate the contributions of specific presynaptic populations, we begin with the spike output files from each simulation in the simulation campaign on the fully connected circuit (Figure 2B ii). Because the spike output files associate each spike in the simulation with the unique ID of the spiking neuron, we can filter out the spikes from the population of interest to create a series of “spike input files” (one per simulation in the original campaign) (Figure 2B iv). We then create a new simulation campaign (Figure 2B v), in which the efferent synapses of the populations other than the populations of interest are artificially activated at the times listed in the “spike input file” (Figure 2B iv-v, blue and green arrows). (Note that this procedure only activates the efferent synapses of these populations, and does not alter their membrane potentials or transmembrane currents) In this new simulation additional spiking activity in this population is not transmitted by efferent synapses (Figure 2B v, greyed-out arrows). We ensure that the outcomes of stochastic processes, such as synaptic release and stochastic ion channels, are preserved between the simulations from the fully connected circuit and those from the disconnected circuit. The SEPs are calculated for the new campaign as described above (we refer to these SEPs as the “decoupled EEG” (Figure 2B vi)), and subtracted from the SEPs from the fully-connected campaign, which we refer to as the “network EEG” (Figure 2B iii). This difference represents the contribution of the presynaptic population to the SEP (Figure 2B vii).

To isolate the contributions of thalamic populations, we replay the unaltered spikes from the fully-connected simulations into a disconnected circuit, without the thalamic input, and subtract the resulting EEG from that of the fully-connected simulation.

In this paper, we isolate the presynaptic contributions of inhibitory neurons in Layer 1, and both excitatory and inhibitory neurons in layers 2/3, 4, 5, and 6, as well as the VPM inputs.

#### Computational requirements

The neural simulations described in this paper were run on the BB5 supercomputer using 32 nodes; each node has two 2.30 GHz, 18 core Xeon SkyLake 6140 CPUs, and 382 GB DRAM. Each simulation requires 2300 core-hours.

#### Comparison with *in vivo* data

To compare the SEPs generated *in silico* with *in vivo* data, we digitized the highest-amplitude SEP recorded on postnatal day 16 in^3^, as the highest-amplitude SEPs were recorded above the somatosensory cortex. The *in vivo* EEG is normalized to its P1 peak.

### Quantification and statistical details

For each *in silico* experiment in the original circuit, 10 trials were conducted, while 9 trials were conducted for each experiment in the SM circuit; each trial corresponds to a different random seed for the background noise injected into all cells. The mean EEG and LFP signals over all trials in the experiment are reported.
