# Supplementary material for: Computational modeling reveals biological mechanisms underlying the whisker flick EEG
Source: iScience. 2025 Oct 17;28(11):113793. doi: 10.1016/j.isci.2025.113793 (PMC12637391; doi:10.1016/j.isci.2025.113793)
Supplement: Document S1. Figures S1–S3 [file mmc1.pdf]

iScience, Volume *28*

Supplemental information

Computational modeling reveals biological mechanisms underlying the whisker flick EEG

Joseph Tharayil, James B. Isbister, Esra Neufeld, and Michael Reimann

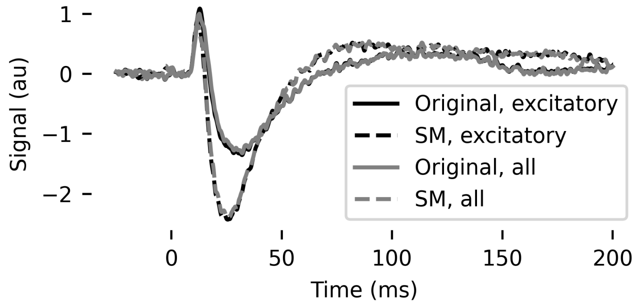

Figure S.1: **Inhibitory cells do not contribute to EEG, Related to Introduction** The contribution to the *in silico* EEG from excitatory postsynaptic cells alone is almost identical to the total EEG, in both the original and the SM circuits. This is expected, as a cell's contribution to the EEG is driven by the difference in the compartment contribution weights between locations of input currents and return currents; this difference is small for interneurons.

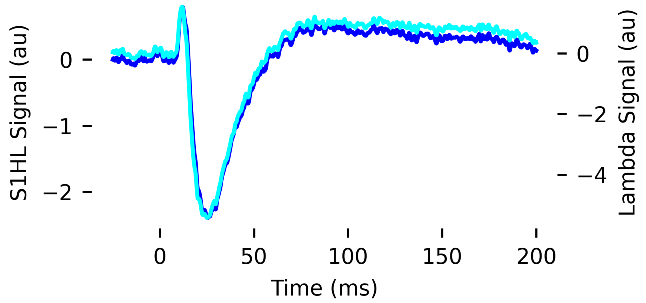

Figure S.2: **Effects of changing reference electrode position, Related to Section 2.2** SEP recorded in the SM circuit from an EEG electrode over the S1FL region, with the reference electrode over the S1HL region or the lambda point. The choice of reference electrode location has little impact on the shape of the SEP. All electrodes are modeled as spheres. For simulations with the S1HL reference, the electrodes have a radius of 0.1 mm; for simulations with the lambda reference, the electrodes have a radius of 0.5 mm.

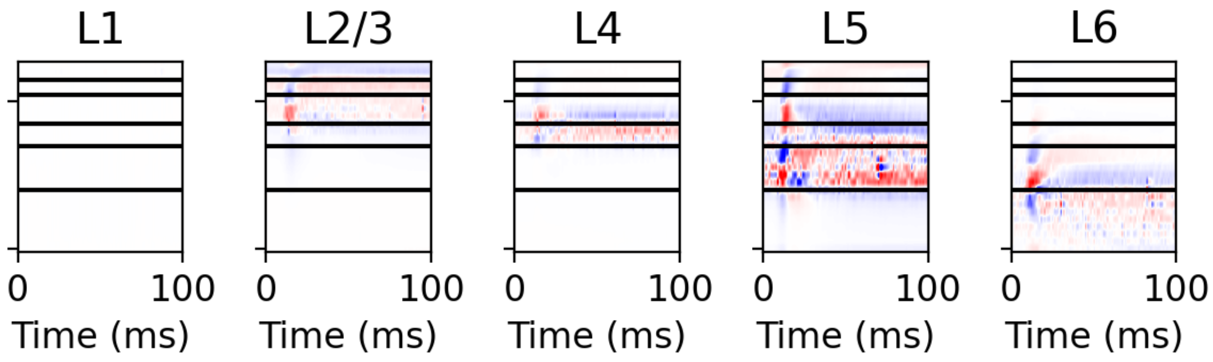

Figure S.3: **Postsynaptic contributions to the CSD from each layer, Related to Section 3.3.1** The CSD is driven primarily by postsynaptic activity in L5 and L6, even for electrodes in supragranualar layers.
